# Outcome and characteristics of COVID-19 patients associated with stroke: a multicenter hospital-based study in Egypt

**DOI:** 10.1186/s41983-022-00517-2

**Published:** 2022-07-07

**Authors:** Ahmed H. Elsheshiny, Hussein Awad El Gharieb, Mostafa Ahmed Sabrh

**Affiliations:** 1 Department of Neurology, Faculty of Medicine, Al-Azhar University, Cairo, Egypt; 2 Department of Internal Medicine, Faculty of Medicine, Al-Azhar University, Cairo, Egypt

**Keywords:** Stroke, Covid-19, SARS-CoV-2, Ischemic stroke, Stroke mortality

## Abstract

**Background:**

Nearly 55 percent of patients are said to be affected by the neurological effects of COVID-19. COVID-19 was shown to be related with stroke in 0.9 to 5% of people. It's critical to assess the impact of COVID-19 on the outcomes of acute ischemic stroke. The goal of this study was to look at the outcomes and characteristics of patients who had an acute ischemic stroke due to covid-19 infection.

**Results:**

The participants in this study were 399 people who had had a stroke. COVID-19 positivity was confirmed in 77 cases, while COVID-19 negativity was confirmed in 322. In the COVID-19 and control groups, the average age of the patients was 65.4 ± 10.2 and 65.3 ± 11.8, respectively. The Covid-19 and control groups had a mean stroke onset of 5.2 ± 2.1 and 5.7 ± 3.8 h, respectively (*P* = 0.12). There was a high in-hospital mortality rate among patients with COVID-19 with a rate of 11.7% compared to 4.04% among the control group (*P* = 0.02). At discharge, the number of patients with mRS > 2 was higher (*P* = 0.001) among the COVID-19. There was a correlation between the mean levels of D-Dimer (*r* = 0.668, *P* < 0.001), the severity of COVID-19 (*r* = 0.802, *P* < 0.001), and mRS > 2.

**Conclusion:**

Despite receiving equal acute care as non-COVID-19 patients, COVID-19 patients had more severe strokes and had worse outcomes. This includes a high chance of death while in the hospital as well as a significant level of disability. Neurologists should use timely and effective therapies, particularly for patients who are at a higher risk of having a stroke**.** This includes elderly patients, patients with severe COVID-19, patients with high levels of D-Dimer, and those with high NIHSS.

## Background

COVID-19 is a novel strain of the -coronavirus family that is enclosed, positive-sense, single-stranded RNA virus. COVID-19 has become a prominent source of illness and mortality worldwide since its emergence in December 2019. The WHO labelled the disease a pandemic in March 2020 [1]. More than 175 million people have been infected with COVID-19 in the year since this date. More than four million people died as a result of the contagiousness, making it the most serious health disaster since the influenza pandemic [2, 3]. According to preliminary estimates, the Middle East accounted for more than 3% of all COVID-19-related death cases worldwide [4–6]. By December 2021, Egypt had confirmed over 380,000 COVID-19 cases, contributing to an estimated $1 billion in revenue, contributing to approximately 21,000 deaths [7].

The respiratory system is most affected by the severe acute respiratory syndrome coronavirus-2 (SARS-CoV-2). Extrapulmonary symptoms like cardiac failure, renal insufficiency, and neurological problems affect a considerable proportion of SARS-CoV-2 patients. Nearly 55 percent of patients are said to be affected by the neurological effects of COVID-19. COVID-19 was shown to be related with stroke in 0.9 to 5% of people [8–10]. This could be related to systemic inflammation and cytokine storm, postinfectious immune-mediated reactions, and direct viral-induced endotheliopathy. With virus particles recovered from numerous tissues, including brain tissue, these diseases may lead to angiopathic thrombosis [11]. Angiotensin-converting enzyme 2 receptors (ACE-2) are abundantly presented within different organs' endothelial and epithelial cells. COVID-19 infection downregulates the expression of these receptors, resulting in a marked increase in blood pressure, volume overload, and progressive hypoxemia [12, 13].

Stroke is linked to a higher rate of mortality in the hospital. Furthermore, it has a significant negative impact on the patient's life and is linked to disability, psychological distress, and a lack of social interactions [14, 15]. It is critical to assess the impact of COVID-19 on the outcomes of acute ischemic stroke. Recognizing such evidence may aid neurologists in better understanding how COVID-19 may affect stroke patients and, as a result, more effectively utilize the most effective therapies for stroke patients. However, there is a scarcity of evidence in the literature. This is due to a dearth of well-structured clinical research, as well as differences in populations, sample bias, heterogeneity, study objectives, and follow-up periods.

These limitations result in controversial results, making it difficult to draw firm conclusions from the available evidence. Therefore, the current study aimed to assess the outcome and characteristics of patients with acute ischemic stroke associated with covid-19 infection.

## Methods

This prospective cohort multicentric study involved 399 patients presented with acute stroke patients diagnosed according to WHO criteria [16]. Patients with or without a diagnosis of COVID-19 infection admitted to our hospitals, during the period from March 2020 to September 2021 were included. Patients with subarachnoid hemorrhage or patients with transient ischemic attack were excluded.

### Ethics approval and consent to participate

After obtaining approval from the local ethics committee, patients who agreed to participate gave their signed informed consent after explanation of the trial benefits and hazards. All procedures were carried out in accordance with the ethical standards of the institutional and/or national research committee and with the 1964 Declaration of Helsinki and its later amendments or comparable ethical standards.

The patients were then divided into two groups. Within four days of admission, the COVID-19 group had a positive nasal SARS-CoV-2 PCR test. Patients who had clinical characteristics indicative of COVID-19 at the time of admission and were determined to be SARS-CoV-2 positive at any point during the first ten days of hospitalization were also included in the study. Patients who were consistently SARS-CoV-2 negative or who were never tested because they did not develop symptoms or evidence of COVID-19 made up the other group. The study excluded patients with traumatic intracerebral hemorrhage, subarachnoid hemorrhage, subdural hematoma, and extradural hematoma.

Demographic data, risk factors, stroke characteristics, management, and stroke severity (assessed using the National Institutes of Health Stroke Scale [NIHSS] score) were recorded. For ischaemic strokes. The TOAST (Trial of ORG 10172 in acute stroke treatment) classification was either taken from the discharge summary or was inferred from the clinical team’s documented assessment of likely stroke aetiology [17]. Stroke territory and revascularization procedures were gathered. The time elapsed between stroke and SARS-CoV-2 infection diagnosis, treatment received, chest computed tomography (CT), and laboratory data were recorded. The in-hospital complications and disability on discharge were determined using the modified Rankin Scale (mRS)**, **which varies from 0 (no symptoms) to 6 (death) [18].

### Statistical analysis

The student's T-test was used to compare continuous normally distributed data in the form of mean and standard deviation (SD). The median and range were used to describe non-normally distributed data, and the Mann–Whitney U test was used to compare between the case and control groups. Categorical variables were reported as a number or a percentage, and groups were compared using Pearson's chi-square test and Fisher's exact test. For non-parametric variables, Spearman's rank correlation coefficient was used to do correlation analysis. The binary logistic regression model was used to determine factors linked to stroke outcomes in COVID-19 patients. The significance is established when two-sides *P*-value < 0.05. Statistical analysis was performed using SPSS software version 25 for Windows (SPSS Inc., Chicago, IL, New York, USA), [19].

## Results

A total of 417 stroke patients were admitted to the emergency department during the study period. Out of them, 77 patients were confirmed SARS-CoV-2 positive (COVID-19 group), and 322 patients were SARS-CoV-2 negative (control group), while 18 patients were excluded from the study. The mean age of the included patients was 65.4 ± 10.2 and 65.3 ± 11.8 in the COVID-19 and control groups, respectively. There were 45 (58.4%) males in the COVID-19 group and 195 (60.6%) males in the control group. Patients with COVID-19 had higher mean levels of total leucocytic count (TLC) (*P* < 0.001), platelet count (*P* < 0.001), and C-reactive protein (CRP) ((*P* < 0.001), in comparison to the control group. There was a statistically significant low mean of total lymphocytic count among COVID-19 group, in comparison to the control group with a mean of 0.86 ± 0.12 and 1.33 ± 0.28, respectively (*P* < 0.0001). There was no difference between age, gender, and comorbidities between both groups (Table 1).

**Table 1:** Comparison between case and control groups regarding baseline factors

Group	Covid-19	Control	*P*-value
	(*N* = 77)	(*N* = 322)	
Variables	Mean ± SD, Number (%)	Mean ± SD, Number (%)	
Age (Years)	65.4 ± 10.2	65.3 ± 11.8	0.94
Gender (Males)	45(58.4%)	195(60.6%)	0.73
Comorbidities			
Obesity	34 (44.2%)	126 (39.1%)	0.42
Smoking	15 (19.5%)	73 (22.7%)	0.54
Diabetes Mellitus	32 (41.6%)	156 (48.4%)	0.28
Hypertension	36 (46.8%)	141 (43.8%)	0.64
History of TIA	11 (14.3%)	41 (12.7%)	0.72
AF	9 (11.7%)	34 (10.6%)	0.77
Stroke history	12 (18.2%)	38 (11.8%)	0.34
Laboratory Findings			
TLC	9.82 ± 2.56	8.11 ± 3.22	< 0.0001
Lymphocytic count	0.86 ± 0.12	1.33 ± 0.28	< 0.0001
Hemoglobin g/dl	12.71 ± 3.09	13.25 ± 2.94	0.15
Platelets count*10.^3^	356.9 ± 76.2	258.8 ± 84.6	< 0.0001
D-dimer mcg/ml	1.64 ± 0.77	0.63 ± 0.21	< 0.0001
CRP mg/dl	87.4 ± 25.4	34.16 ± 7.81	< 0.0001
Levels of oxygen therapy			
Room air	36 (46.7%)	290 (90%)	< 0.0001*
Low-flow oxygen	19 (24.7%)	16 (4.97%)	
High-flow oxygen	13 (16.9%)	9 (2.8%)	
Non-invasive CPAP	1 (1.3%)	2 (0.62%)	
Invasive ventilation	8 (10.4%)	5 (1.55%)	

*TIA* Transient Ischemic Attack, *AF* Atrial Fibrillation, *TLC* total leukocytic count, *CRP* C-reactive protein

Regarding Stroke characteristics and treatment, the mean stroke onset was 5.7 ± 3.8 and 5.2 ± 2.1 h among the Covid-19 and control groups (*P* = 0.12). On admission, 42 (54.5%) patients were treated with antiplatelets among the Covid-19 group, relative to 174 (54%) patients within the control group (*P* = 0.94). Furthermore, 12(15.6%) and 59(18.3%) patients received Alteplase among the Covid-19 and control groups (*P* = 0.62), respectively. The most frequently affected territory was the anterior circulation, affecting 46 (59.74%) and 218 (67.7%) patients within the Covid-19 and the control groups (*P* = 0.52). The cause of the stroke was large vessel occlusion in 21(27.3%) patients within the Covid-19 group and 68(21.1%) patients within the control group (*P* = 0.69). although percentage of patients with fits in Covid-19 cases was higher than control group yet P value was non-significant (0.07) (Table 2).

**Table 2:** Comparison between case and control groups regarding procedures and outcomes

Groups	Covid-19	Control	*P*-value
	(N = 77)	(*N* = 322)	
Variables	Mean ± SD, Number (%)	Mean ± SD, Number (%)	
Interventions on admission			
Thrombectomy	3 (3.9%)	15 (4.7%)	0.99
Alteplase	12 (15.6%)	59 (18.3%)	0.62
Antiplatelet	42 (54.5%)	174 (54%)	0.94
Anticoagulant	10 (13%)	21 (6.5%)	0.09
Stroke onset hours	5.7 ± 3.8	5.2 ± 2.1	0.12
Stroke territory			
Anterior circulation	46 (59.74%)	218 (67.7%)	0.52
Posterior circulation	24 (31.1%)	76 (23.6%)	
Multiple territory	7(9.1%)	28(8.7%)	
Stroke mechanism			
Small arteries	19 (24.7%)	86 (26.7%)	
Large vessels	21 (27.3%)	68 (21.1%)	0.69
Cardio-embolic	3 (3.9%)	12 (3.7%)	
Other	34 (44.2%)	156 (48.4%)	
Fits with stroke	9 (11.7%)	17 (5.3%)	0.07

Concerning the impact of COVID-19 on the stroke outcomes, there was no significant difference in disease severity between the COVID-19 and control groups at admission (*P* = 0.906). Within the COVID-19 and control groups, the median NIHSS was 7 (0–42) and 12 (1–42), respectively. Patients with COVID-19 had a higher in-hospital death rate of 11.7 percent compared to 4.04 percent in the control group (*P* = 0.02). The most common cause of death in the Covid-19 group was respiratory failure and pulmonary embolism, accounting for 4 (5.19%) and 3 (3.89%) patients, respectively. At discharge, the number of patients with mRS > 2 was statistically substantially greater (*P* = 0.001) in the COVID-19 group, with 50 (64.93%) patients compared to 133 (41.3%) patients in the control group (Table 3).

**Table 3:** The impact of COVID-19 on the stroke outcomes

Groups	Covid-19	Control	*P*-value
	(*N* = 77)	(*N* = 322)	
Variables	Number (%), Median (Range)	Number (%), Median (Range)	
NIHSS	7 (0–42)	12 (1–42)	0.906
IQR	8.5	11	
mRS > 2	50 (64.93%)	133 (41.3%)	0.001
In-hospital death	9 (11.7%)	13(4.04%)	0.02
Respiratory failure	4 (5.19%)	0 (0%)	
Sepsis	0 (0%)	5 (1.55%)	
Aspiration pneumonia	0 (0%)	2 (0.62%)	
Pulmonary embolism	3 (3.89%)	2 (0.62%)	
Myocardial infarction	0 (0%)	1 (0.31%)	
Acute kidney injury	2 (2.59%)	0 (0%)	

*MRS* The modified Rankin Scale, *NIHSs* National Institutes of Health Stroke Scale

**Fig. 1:**
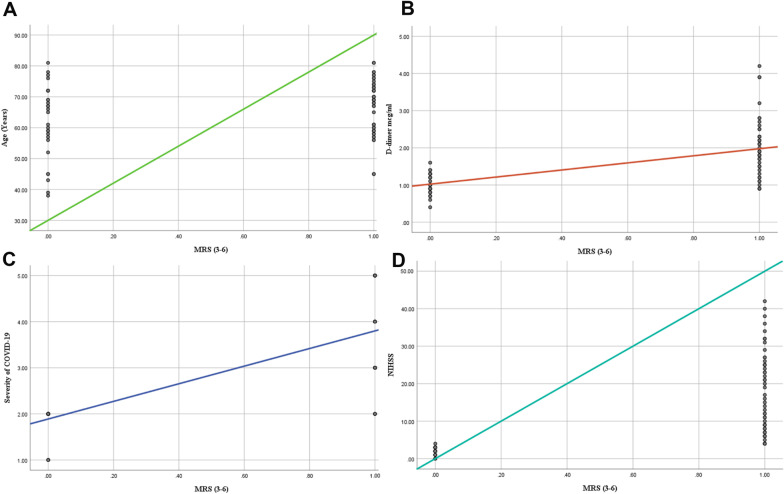
Scatter plots with regression lines showing the association between Modified Rankin Scale of 3–6 grade among patients with COVID-19 and **A** Patient’s age (Years) **B** D-dimer mcg/ml **C** Severity of COVID-19 Symptoms, **D** National Institutes of Health Stroke Scale for severity of stroke at admission

As for Predictors of stroke outcomes in COVID-19 Patients, the age of the patients and mRS > 2 had a positive connection (*r* = 0.227, *P* = 0.047). There was an association between mean D-Dimer levels (*r* = 0.668, *P* = 0.001), COVID-19 severity (*r* = 0.802, *P* = 0.001), NIHSS (*r* = 0.826, *P* = 0.001), and mRS > 2 in this regard. Neither the NIHSS (*P* = 0.966) nor the severity of COVID-19 (*P* = 0.978) were significant predictors of mRS > 2 at discharge in the binary logistic regression model (Tables 4, 5 and Fig. 1).

**Table 4:** Correlation between COVID-19 related data and outcomes at discharge

	The modified Rankin Scale > 2	
Variables	*r*	*P*-value
Age (Years)	0.227	0.047
D-dimer mcg/ml	0.668	< 0.001
CRP	− 0.155	0.178
Severity of COVID-19	0.802	< 0.001
NIHSS	0.826	< 0.001

*CRP* C-reactive protein, *NIHSs* National Institutes of Health Stroke Scale, *r* Correlation Coefficient

**Table 5:** Binary logistic regression model for factors associated with COVID-associated stroke outcomes

Variables	*β*	Standard error	P-value
NIHSS	40.258	942.310	0.966
D-dimer mcg/ml	54.455	1629.756	0.973
Severity of COVID-19	− 80.866	2874.075	0.978

*NIHSs* National Institutes of Health Stroke Scale

## Discussion

The present COVID-19 situation has highlighted the importance of determining the pandemic's impact on healthcare [20]. While several research have looked at the relation between COVID-19 and neurological symptoms, the influence of the present pandemic on stroke outcomes has to be looked into more. This is because variances in the investigated population, healthcare services, and study technique make it difficult to draw a final conclusion from the existing literature [21, 22]. As a result, the current prospective study was carried out to evaluate the short-term outcomes of stroke in Egyptian patients diagnosed with COVID-19 and to identify potential determinants of stroke outcomes in the short-term.

COVID-19 patients experienced poor stroke outcomes, according to the current study. In patients with COVID-19, the in-hospital death rate was around three times that of the control group. At patients discharge, most COVID-19 patients had poor stroke outcomes, with a risk of roughly 1.5 times that of COVID-19 patients. Furthermore, patients who were elderly, had severe COVID-19, had high levels of D-Dimer, or had a high NIHSS had a higher chance of bad stroke outcomes at discharge. In the binary regression model, however, neither of these characteristics was significantly linked with mRS > 2. COVID-19 has been linked to the onset of ischemic stroke via thrombotic and inflammatory processes [23, 24].

The development of a cytokine storm and activation of the innate immune system, embolic events propagated by pre-existing or new-onset arrhythmias, and hypoxia-induced ischemia secondary to severe pulmonary illness are among the primary hypothesized processes. These conditions exacerbate the effects of stroke, resulting in additional neurological impairments and, as a result, poor outcomes. These findings matched those of Mart-Fàbregas and colleagues, published in 2021. They discovered that patients who had an ischemic stroke linked to COVID-19 had a more severe stroke. In comparison to patients without COVID-19, they reported a 3.1-fold increase in mortality risk within 72 h of admission [25]. The significant in-hospital death rate linked with COVID-19 is due to the concomitant respiratory distress and multiorgan failure, in addition to the already weakened general state due to the stroke.

This finding can help epidemiologists and doctors treating stroke on the front lines, especially in places where COVID-19 prevalence is high [24]. COVID-19 plays a varied role in stroke risk depending on the severity of the illness. Patients with more serious conditions are more likely to have a stroke and have poorer results. The fact that stroke occurred in 5.7 percent of patients with critical illness compared to 0.8 percent of individuals with milder COVID-19 condition backs up this claim [25]. In hospitalized patients with severe disease during the COVID-19 outbreak in Wuhan, China, a retrospective analysis found an incidence of acute ischemic stroke of about 5% [26]. Our main concern since the introduction of COVID-19 in Egypt has been to maintain the same level of efficiency in the acute stroke service.

According to the findings of the current study, some stroke victims avoided obtaining emergency care in hospitals for fear of infection. This explains why there are fewer hospitalized stroke patients and a higher NIHSS score, which indicates a severe stroke. Patients having mild strokes during the COVID phase may be less likely to seek medical care [27, 28]. Roushdy and colleagues in 2020 studied 93 Egyptian patients with acute ischemic stroke. They found that concern of contracting COVID-19 and a lack of healthcare resources prevented 11 percent of patients from receiving prompt stroke treatment [29]. In this regard, Aref and colleagues in 2021 reported that in the COVID-19 era, fear of infection and lockdown issues were the most common causes of delayed arrival in ischemic stroke [30].

When compared to earlier findings on stroke in COVID-19 patients, the new study had several advantages. It was based on a multicenter hospital registry to begin with. COVID-19 patients' outcomes were compared to non-COVID-19 patients who had an acute stroke and were treated with the same management procedures. These protocols should be tailored to the new in-hospital pathways created to prevent pandemic viral spread. Other investigations, like as chest CT, had an impact on stroke care time metrics in all patients admitted during the pandemic. The current study, on the other hand, has some drawbacks. To begin with, a variety of circumstances could have influenced whether patients sought emergency medical attention, were admitted to a hospital, and received extensive treatment. Some COVID-19 and ischemic stroke patients may have died before reaching the hospital, while others with lesser symptoms or treatment limits may have remained at home. This could have resulted in an underestimation of the total rate of ischemic stroke in COVID-19 patients. In addition, data from a registry set up to detect cardiac and thromboembolic problems in COVID-19 patients was used in part of our retrospective investigation.

## Conclusion

Despite receiving equal acute care as non-COVID-19 patients, COVID-19 patients had more severe strokes and had worse outcomes. This includes a high chance of death while in the hospital as well as a significant level of disability. Neurologists should use timely and effective therapies, particularly for patients who are at a higher risk of having a stroke. This includes elderly patients, patients with severe COVID-19, patients with high levels of D-Dimer, and those with high NIHSS. Further studies with adequate sample size and a more extended follow-up period are required to tackle the current study's limitations.

## Data Availability

The datasets analysed during the current study are not publicly available as the participants requested that, but are available from the corresponding author on reasonable request.

## Abbreviations

ACE-2: Angiotensin-converting enzyme 2 receptors
mRS: Modified Rankin Scale
SARS-CoV-2: The severe acute respiratory syndrome coronavirus-2
PCR: Polymerase chain reaction
TLC: Total leucocytic count
CRP: C-Reactive protein
NIHSS: The National Institutes of Health Stroke Scale
